# *TET2/IDH1/2/WT1* and *NPM1* Mutations Influence the *RUNX1* Expression Correlations in Acute Myeloid Leukemia

**DOI:** 10.3390/medicina56120637

**Published:** 2020-11-24

**Authors:** Sergiu Pasca, Ancuta Jurj, Ciprian Tomuleasa, Mihnea Zdrenghea

**Affiliations:** 1 Department of Hematology, Iuliu Hatieganu University of Medicine and Pharmacy, 400012 Cluj Napoca, Romania; ciprian.tomuleasa@gmail.com (C.T.); mzdrenghea@umfcluj.ro (M.Z.); 2 Medfuture Research Center for Advanced Medicine, Iuliu Hatieganu University of Medicine and Pharmacy, 400349 Cluj Napoca, Romania; 3 Research Center for Functional Genomics, Biomedicine and Translational Medicine, Iuliu Hatieganu University of Medicine and Pharmacy, 400337 Cluj-Napoca, Romania; ancajurj15@gmail.com; 4 Department of Hematology, Ion Chiricuta Clinical Cancer Center, 400124 Cluj Napoca, Romania

**Keywords:** acute myeloid leukemia, *RUNX1*, *NPM1*, *TET2*

## Abstract

*Background and objectives:* Mutational analysis has led to a better understanding of acute myeloid leukemia (AML) biology and to an improvement in clinical management. Some of the most important mutations that affect AML biology are represented by mutations in genes related to methylation, more specifically: *TET2*, *IDH1*, *IDH2* and *WT1*. Because it has been shown in numerous studies that mutations in these genes lead to similar expression profiles and phenotypes in AML, we decided to assess if mutations in any of those genes interact with other genes important for AML. *Materials and Methods:* We downloaded the clinical data, mutational profile and expression profile from the TCGA LAML dataset via cBioPortal. Data were analyzed using classical statistical methods and functional enrichment analysis software represented by STRING and GOrilla. *Results:* The first step we took was to assess the 196 AML cases that had a mutational profile available and observe the mutations that overlapped with *TET2/IDH1/2/WT1* mutations. We observed that *RUNX1* mutations significantly overlap with *TET2/IDH1/2/WT1* mutations. Because of this, we decided to further investigate the role of *RUNX1* mutations in modulating the level of *RUNX1* mRNA and observed that *RUNX1* mutant cases presented higher levels of *RUNX1* mRNA. Because there were only 16 cases of *RUNX1* mutant samples and that mutations in this gene determined a change in mRNA expression, we further observed the correlation between *RUNX1* and other mRNAs in subgroups regarding the presence of hypermethylating mutations and *NPM1*. Here, we observed that both *TET2/IDH1/2/WT1* and *NPM1* mutations increase the number of genes negatively correlated with *RUNX1* and that these genes were significantly linked to myeloid activation. *Conclusions:* In the current study, we have shown that *NPM1* and *TET2/IDH1/2/WT1* mutations increase the number of negative correlations of *RUNX1* with other transcripts involved in myeloid differentiation.

## 1. Introduction

In the last decades, acute myeloid leukemia (AML) has seen an important increase in the use of mutational analysis. This has led to a better understanding of the biology of this disease and, added to this, several hospitals have employed the clinical use of mutational panel for AML, influencing both prognosis and therapeutic management [1]. Nonetheless, there are still more studies to be done so that AML could be completely understood and properly classified in subentities.

Of the mutations that have been shown to be important in AML biology, of particular interest are mutations in *TET2*, *IDH1*, *IDH2* and *WT1*, as they are known to influence both the epigenetic and transcriptomic landscape of AML [2]. These mutations are considered as having similar effects regarding the methylation changes, transcriptomic profile and immature phenotype they induce. These effects revolve around the loss of function of the TET2 enzyme. This enzyme catalyzes the oxidation of methyl cytosine to hydroxy methyl cytosine and then takes part in the further sequential oxidation steps of the hydroxy methyl to formil and carboxyl groups, the latter, alongside the cytosine it is attached to being excised and replaced with an unmethylated cytosine [3]. Mutations in *TET2* generally lead to the formation of a nonfunctional form of the enzyme, which is unable to catalyze the abovementioned process, leading to the hypermethylation of certain DNA loci [3]. In the case of IDH1 and IDH2, it has been observed that their mutations lead to a gain of function effect that converts the activity of these enzymes from generating α-keto glutarate to generating 2-hydroxy glutarate, which acts as a competitive inhibitor of TET2, thus reducing its activity and leading to a similar effect as in the case of *TET2* mutations [4]. Moreover, WT1 has been shown to physically interact with TET2 and to be necessary for TET2 activity on methylated cytosines. Mutations in *WT1* have been observed to generate a nonfunctional form of WT1 and to subsequently impair the effects of normal TET2 activity [5].

We, as well as other authors, have shown that, in the case of AML, these mutations have a similar methylation and transcription profile and are associated with more immature forms of AML. Because of this, it can be proposed that these mutations may influence the maturity of AML blasts and potentially form a separate entity of AML [2,6,7].

Further on, we will refer to *TET2*, *IDH1*, *IDH2* and *WT1* mutations as hypermethylating mutations because of the effect that mutations in these genes have on the overall methylation.

Because, as mentioned, the hypermethylating mutations were shown to form one entity in AML, we decided to further investigate if this entity would have specific interactions with gene mutations or expression profiles that are regarded as important for AML.

## 2. Material and Methods

The results shown here are based upon data generated by the TCGA Research Network: https://www.cancer.gov/tcga. The RNAseq z scores, clinical data and mutational profile from the LAML TCGA cohort were downloaded via cBioPortal [8,9].

Data analysis was performed using R 4.0.1 (R Foundation for Statistical Computing, Vienna, Austria). Contingency tables were analyzed using Fisher’s test. Normality of the distribution was assessed using Shapiro–Wilk’s test and histogram visualization. Spearman’s test was used to determine the association between two non-normally distributed variables. The Benjamini–Hochberg (BH) method was used for adjusting the *p* value in the case of multiple analyses. Gene Ontology [10] was used as the source for the biological processes. STRING was used to visualize the gene networks [11]. GOrilla was used to visualize the links between biological processes [12]. A *p* value under 0.05 was considered statistically significant.

## 3. Results

**Figure 1:**
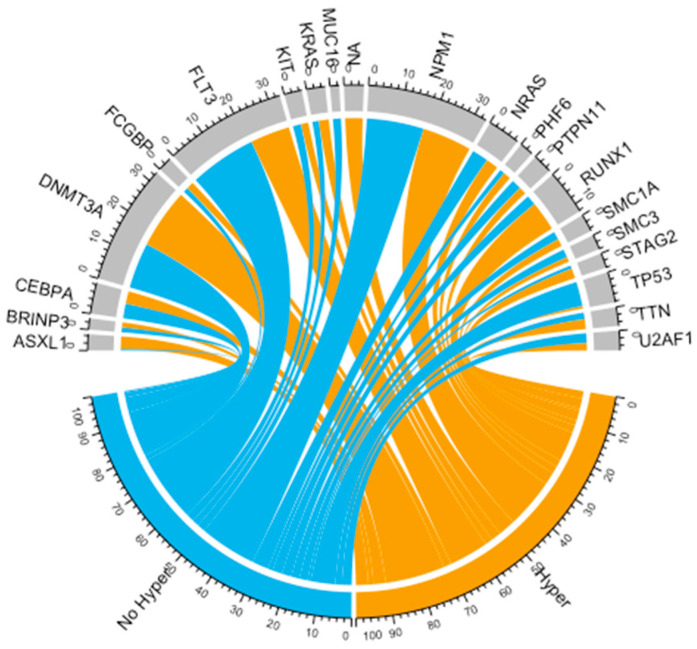
Chord diagrams showing the association between hypermethylating mutations and other mutations commonly occurring in acute myeloid leukemia (AML) in absolute counts.

Our first approach was to assess the 196 LAML TCGA samples that had available mutational profile regarding the overlaps that hypermethylating mutations have with other gene mutations (Figure 1). The overlaps in relative counts of these mutations have been presented in Supplementary Figure S1.

Of the 196 cases, 63 presented any of the hypermethylating gene mutations, with 5 cases presenting two of these mutations co-occurring. Of those, in 4 out of the 5 cases, one of the mutations occurred in *IDH1*. We observed that hypermethylating mutations overlapped with 11 (64.7%) of the *RUNX1* mutated samples. Because of this, we decided to further investigate the dynamics between hypermethylating mutations and *RUNX1* mutations. It has to be mentioned that we did not take into consideration *RUNX1* fusions but only single nucleotide polymorphisms and indels (insertions and deletions). Of the 196 sequenced LAML samples, 17 presented *RUNX1* mutations. In Table 1, we present the importance of the associations between hypermethylating mutations and other mutations occurring in AML.

*RUNX1* mutations were in part almost exclusively of the intermediate cytogenetic risk, with 16 cases having intermediate cytogenetic risk and 1 not determined (*p* < 0.01). Of the 16 cases, 8 had normal karyotype and 8 had intermediate risk cytogenetic abnormalities. The description of *RUNX1* mutations is offered in Supplementary Table S1. Effect of different mutations was inferred from the study of Li et al. [13].

**Figure 2:**
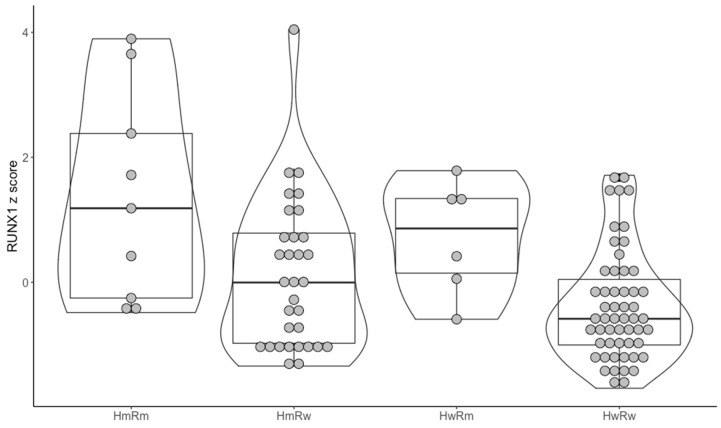
*RUNX1* expression considering the hypermethylating and *RUNX1* mutation overlaps. Hm = hypermethylating mutation; Rm = *RUNX1* mutation; Hw = hypermethylating wild type; Rw = *RUNX1* wild type.

The hypermethylating group was also associated with intermediate cytogenetic risk (*p* < 0.001). Because of this, we decided to further analyze only samples with intermediate cytogenetic risk. Thus, of the 115 intermediate cytogenetic risk AML samples, 101 also had RNAseq data available. Of the 101, 9 had both one of the hypermethylating mutations and a *RUNX1* mutation, 32 had only a hypermethylating mutation, 6 had only *RUNX1* mutations and 54 had none of the selected mutations. The general characteristics of the selected patients is presented in Supplementary Table S2. After this, we wanted to see if there were any differences regarding the *RUNX1* expression between the mentioned groups. There was a statistically significant difference between the 4 groups (*p* < 0.01). For the post hoc test, we used the pairwise Mann–Whitney–Wilcoxon rank sum test with Benjamini–Hochberg adjustment. The ones that reached statistical significance were HwRw (hypermethylator wild type and *RUNX1* wild type) versus HmRm (hypermethylator mutated and *RUNX1* mutated) (*p* < 0.01) and HwRw versus HwRm (hypermethylator wild type and *RUNX1* mutated) (*p* = 0.04). In this case, RUNX1 mutations were associated with a higher *RUNX1* mRNA expression (Figure 2).

**Figure 3:**
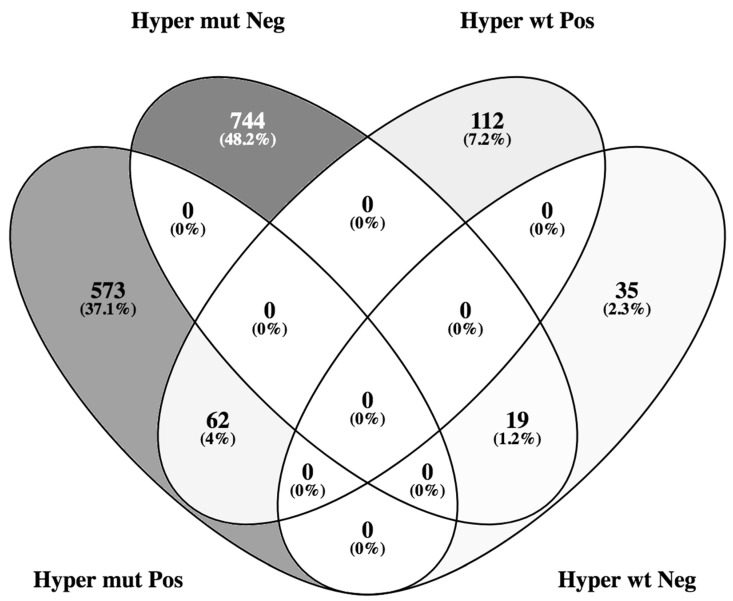
Venn diagram of the overlaps between genes that were significantly positively or negatively correlated with *RUNX1* considering hypermethylating mutation or wild type. Hyper mut = hypermethylating mutation; Hyper wt = hypermethylating wild type; Pos = positive correlation with RUNX1; Neg = negative correlation with *RUNX1*.

After this, we wanted to observe if *RUNX1* mRNA expression correlates with the expression of other genes and if hypermethylating mutations influence these correlations. We filtered the correlations with a BH *p* adjusted value < 0.05 and an absolute rho correlation coefficient over 0.5. When no subgrouping based on the hypermethylating status was done, there were 439 altered genes, of which 275 positively correlated and 164 were negatively correlated. We observed that there was a significant number of processes related to myeloid immunity. When analyzing only the hypermethylating mutated group, there were 1398 altered genes of which 635 were positively correlated and 763 negatively correlated. Conversely, when analyzing the hypermethylating wild-type group, there were 228 altered genes, of which 174 positively correlated and 54 negatively correlated. What can be observed is that hypermethylating mutations increase the number of correlations that *RUNX1* presents (Figure 3).

**Figure 4:**
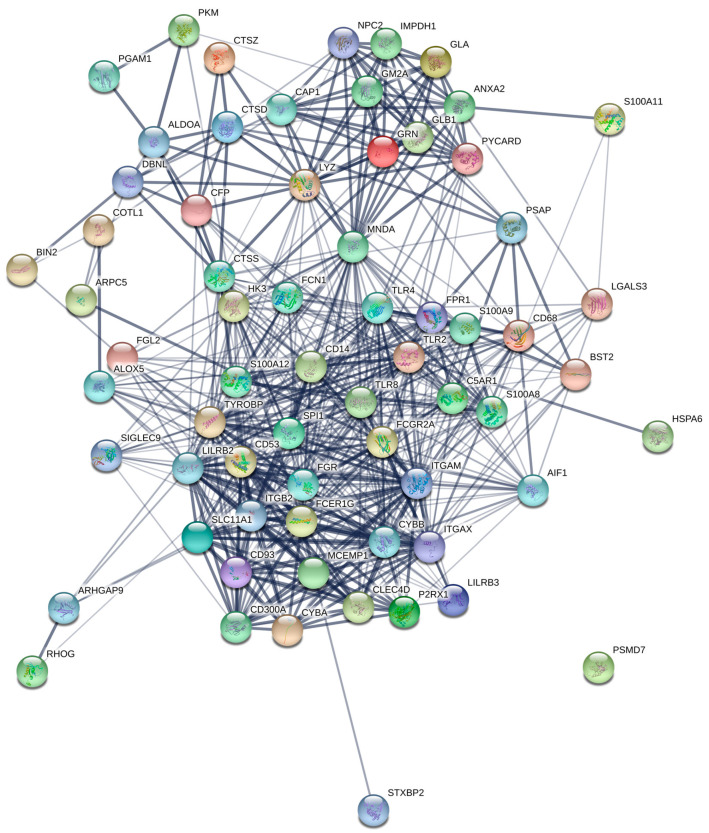
STRING diagram for the genes related to myeloid activation observed to be negatively correlated with *RUNX1* expression when hypermethylating mutations were present.

Because there were a significant number of genes negatively correlated with the *RUNX1* transcript that were related to myeloid activation, we represented the network formed by these genes in Figure 4.

**Figure 5:**
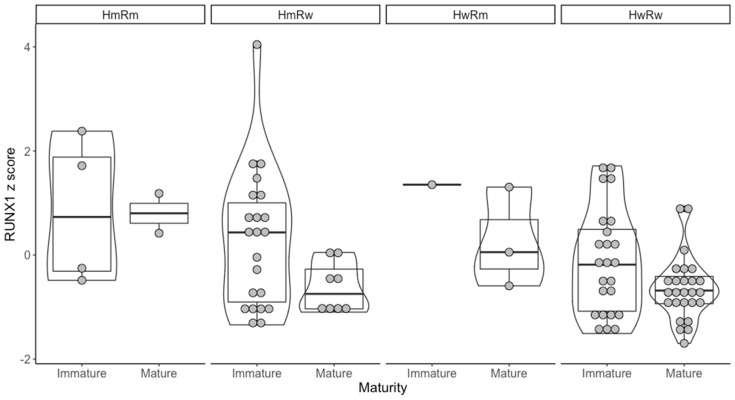
*RUNX1* expression differences between AML with different maturity.

Because of this role in myeloid activation, we further wanted to offer an extension of Figure 1 in which to show the different *RUNX1* expression between mature (M4/5) and immature (M1/2) AML (Figure 5).

We observed that, overall, there was a higher expression of *RUNX1* in immature AML (*p* = 0.015). Additionally, there was a higher expression of *RUNX1* in Rm mature compared to Rw mature (*p* < 0.01).

**Figure 6:**
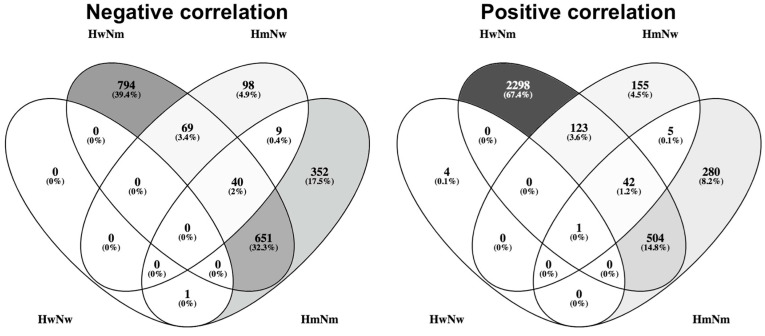
Venn diagrams representing the effects of hypermethylating and *NPM1* mutation overlaps on *RUNX1* associations. We represented one Venn diagram only considering the negative correlations and the other only considering the positive correlations. Hw = hypermethylating gene wild type; Hm = hypermethylating gene mutation; Nw = *NPM1* wild type; Nm = *NPM1* mutation.

Another gene that is known to interact with *RUNX1* is *NPM1*. Mutations in *NPM1* have been shown to change the location of the NPM1 protein from the nucleus to the cytoplasm, taking with it the transcription factor SPI1, an important transcription for myeloid development. Because of this, *NPM1* mutations lead to a decoupling of differentiation from proliferation in myeloid blasts [14]. Thus, we decided to assess what would be the effects of the overlap between hypermethylating mutations and *NPM1* mutations regarding *RUNX1* correlations. In doing so, we observed that most negatively correlated genes were in the HwNm and HmNm groups. Added to this, HwNm presented an important number of the positively correlated genes (Figure 6).

We further assessed the influence that HmNw and HmNm have on myeloid activation considering either positive or negative correlation with *RUNX1*. Thus, HmNw presented 35 genes related to myeloid activation, while HmNm presented 166 genes related to myeloid activation. When assessing the processes of the negatively correlated genes in HmNm, we observed that there were 157 genes related to myeloid activation, while in the case of positive correlation, myeloid activation was not observed as a significant biological process. Interestingly, this effect was still present in the case of HwNm negative correlation, where we observed 159 genes related to myeloid activation.

**Figure 7:**
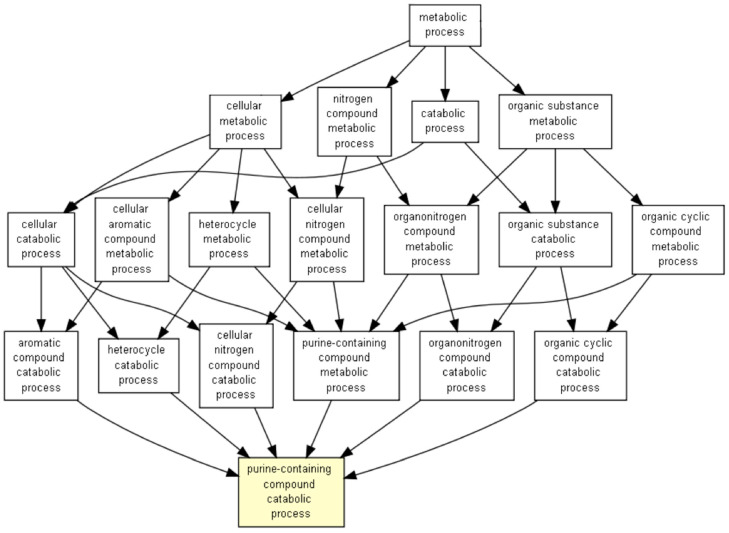
Representation of HwNm positively correlated processes.

Interestingly, there were an impressive number of positively correlated genes in the case of HwNm, and because of the high number of genes altered, we represented the altered biological processes using the GOrilla software (Figure 7).

After the GOrilla analysis, we observed that the main altered biological processes were related to purine metabolism.

## 4. Discussions

In the current study, we have shown that *RUNX1* mRNA correlations are altered by mutations occurring in *NPM1* and *TET2/IDH1/2/WT1*. Interestingly, we observed that the number of genes negatively correlated with *RUNX1* transcript were increased by these mutations. Interestingly, we observed that most of these mutations influence *RUNX1* DNA binding. More than this, the most frequent hotspot mutations, as seen in Supplementary Table S1, were located at residues R135 and R174. Our observations regarding the increase in *RUNX1* expression could be accounted for either because of a compensatory increase in transcription by the normal allele, the advantageous selection of *RUNX1* mutated cells with a higher *RUNX1* mutated transcript or because of the general changes that *RUNX1* mutations induce that ultimately also lead to *RUNX1* overexpression.

Moreover, the negatively correlated genes were observed to be linked to myeloid activation. This observation might add another argument to the importance of several genes like *TET2/IDH1/2/WT1*, *NPM1* and *RUNX1* in myeloid activation, as we and other groups have shown that the immature phenotype of AML is associated with *TET2/IDH1/2/WT1* mutations [2,3]. This is also supported by the fact that *RUNX1* has been shown to induce the expression of *IL3* and *SPI1*, which are known for their importance in myeloid differentiation [15]. Haploinsufficiency of *RUNX1* has been shown to induce the alteration of hematopoietic stem cell formation [16], with other studies showing that RUNX1 induces the transcription of *IL-3* with importance in the hematopoietic stem cell generation in the aorta-gonad mesonephros region, yolk sac and placenta. Moreover, when IL-3 is replaced in the case of *RUNX1* haploinsufficiency, the generation of hematopoietic stem cells was shown to be enhanced [17]. Added to this, IL-3 can not only have an effect in immature myeloid cells, but also in mature ones [18]. This role is important considering stress response of myeloid cells as in the case of sepsis [19], or in the atherogenesis process, as IL-3 stimulates monocytes infiltrating the atherosclerotic plaques [20]. Because of the implication that IL-3 has in stimulating myeloid cells, it is of no surprise that, in AML, IL-3R is of real importance as its upregulation was associated with blast proliferation and reduced overall survival [21,22,23]. Thus, it is possible that the interaction between RUNX1 and IL-3 might have an importance in determining the activity of myeloid blasts and could represent a valuable hypothesis for further studies.

Additionally, it has been observed that the TET2, RUNX1, SPI1, NPM1 complex is disrupted by mutant NPM1, which leads to the translocation of the NPM1 protein to the cytoplasm along with the transcription factor SPI1, leading to an impairment in myeloid differentiation [14]. Moreover, it has been shown that RUNX1 interacts with TET2 and amplifies the demethylating properties of TET2, influencing hematopoietic stem cell development [24]. Another argument for the importance of RUNX1 in myeloid differentiation has been shown in a murine model in which it has been observed that RUNX1 is necessary for hematopoietic lineage commitment [25].

In addition to the role of RUNX1 in myeloid activation, we have also observed that *NPM1* mutations associated with *TET2/IDH1/2/WT1* wild type determine *RUNX1* to be positively correlated with genes related to purine metabolism. Because of this, it can be hypothesized that purine analogs used in AML, like fludarabine and cladribine, could have a different effect on AML based on the mutational status of *TET2/IDH1/2/WT1* and *NPM1* and on the expression level of *RUNX1*. There is an important possibility that this is the case, as *NPM1* is known to offer a good prognosis in AML [26]. Fludarabine and cladribine are used in the clinical scenario as components of FLAG (fludarabine, cytarabine, granulocyte colony stimulating factor) and of CLAG (cladribine, cytarabine, granulocyte colony stimulating factor), respectively. This is generally the case as the combination between these purine analogues and cytarabine are used to this day because of the synergy between these agents [27]. Most frequently, these regimens have been used in the case of relapse/refractory AML representing viable therapeutic options [28,29,30,31]. Between fludarabine- and cladribine-containing regimens used for relapsed/refractory AML, the complete remission rates, overall survival and relapse free survival are similar. Nonetheless, it has to be mentioned that cladribine-based regimens were shown to be better in patients with favorable prognostic factors like nonpoor cytogenetic risk and the presence of complete remission at the first induction therapy [29]. Thus, these agents might have different efficacy based on disease characteristics. Currently, *NPM1* mutations are known for their favorable prognosis role and have been included in the stratification of the World Health Organization from 2016 or myeloid neoplasms. It has to be considered that these conclusions were taken from AML studies using intensive chemotherapy regimens [26,32]. Interestingly, Kantarjian has mentioned that *NPM1* mutations might be associated with cladribine sensitivity [33]. More than this, a regimen combining cladribine with low-dose cytarabine alternating with decitabine has been shown to induce either complete remissions or complete remissions with incomplete count recovery in all patients presenting with an *NPM1* mutation [34]. Further trials could assess if different combinations between mutational statuses of *NPM1* and *TET2/IDH1/2/WT1* and *RUNX1* expression level have an impact in predicting cladribine or fludarabine sensitivity.

In future studies, we plan to further assess other transcription factors that colocalize with TET2 to determine what would be the effects of the interactions. Nonetheless, for such a study, a larger cohort would be necessary. Some of the important transcription factors that have been shown to colocalize with TET2 are represented by, but not limited to, NANOG [35], EBF1 [36], PPARγ [37] and IDAX [38].

The main limitation of this study is represented by the fact that it has been performed on an online dataset without the use of other experimental data. Nonetheless, we could argue that the results presented in this manuscript add useful information regarding the interactions between *NPM1* and *TET2/IDH1/2/WT1* mutations and the correlations that *RUNX1* presents could lead to a better understanding of myeloid differentiation and, potentially, the formation of novel AML subtypes that would be treated in a personalized manner.

## 5. Conclusions

In the current study, we have shown that *NPM1* and *TET2/IDH1/2/WT1* mutations increase the number of negative correlations of *RUNX1* with other transcripts, especially with transcripts involved in myeloid differentiation. The results shown here would not only reveal more insights in the biology of AML maturation, but could also lead to better therapeutic management strategies tailored to each AML subtype.

## Figures and Tables

**Table 1:** The association between hypermethylating mutations and other mutations. OR = odds ratio; CI = confidence interval.

Mutated Gene	OR	Lower 95% CI	Upper 95% CI	*p*-Value
*TP53*	0.097	0.002	0.659	0.006
*RUNX1*	3.428	1.089	11.961	0.032
*FLT3*	0.482	0.221	1.014	0.043
*ASXL1*	6.904	0.663	346.497	0.068
*MUC16*	0	0	1.779	0.158
*STAG2*	3.431	0.475	39.006	0.200
*DNMT3A*	1.355	0.646	2.820	0.387
*TTN*	1.689	0.303	9.430	0.477
*KRAS*	1.689	0.303	9.430	0.477
*PHF6*	1.678	0.218	12.937	0.674
*U2AF1*	0.537	0.051	3.129	0.711
*SMC1A*	0.651	0.060	4.126	0.711
*SMC3*	0.651	0.060	4.126	0.711
*NPM1*	0.852	0.407	1.753	0.734
*CEBPA*	0.717	0.154	2.712	0.768
*NRAS*	0.811	0.207	2.764	0.787
*PTPN11*	0.818	0.128	4.000	1
*KIT*	0.990	0.148	5.299	1
*FCGBP*	1.103	0.090	9.915	1
*BRINP3*	1.103	0.090	9.915	1

## Supplementary Materials

The following are available online at https://www.mdpi.com/1010-660X/56/12/637/s1, Figure S1. Chord diagram presenting the overlaps in relative counts between hypermethylating mutations and other mutations frequently occurring in AML. Table S1. Table of the *RUNX1* mutations occurring in the samples presenting intermediate cytogenetic risk. Table S2. Summary table of the cohort analyzed included for the assessment of *RUNX1* transcript correlations.
