# Supplementary figures and images for: TET2/IDH1/2/WT1 and NPM1 Mutations Influence the RUNX1 Expression Correlations in Acute Myeloid Leukemia
Source: Medicina (Kaunas). 2020 Nov 24;56(12):637. doi: 10.3390/medicina56120637 (PMC7760270; doi:10.3390/medicina56120637)

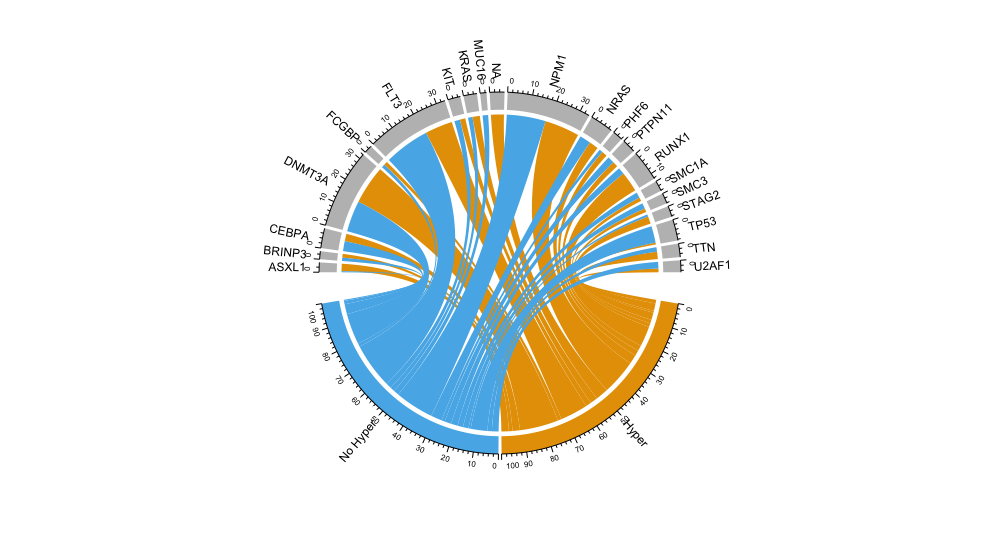

Supplement: Supplementary file 1 [file medicina-56-00637-s001.zip › Supplementary FIgure 1.tiff]
